# Psoas Minor Muscle: A Cadaveric Morphometric Study

**DOI:** 10.7759/cureus.2447

**Published:** 2018-04-08

**Authors:** Pamela Dragieva, Mihaela Zaharieva, Yordan Kozhuharov, Krasimir Markov, George S Stoyanov

**Affiliations:** 1 Student, Faculty of Medicine, Medical University – Varna “Prof. Dr. Paraskev Stoyanov”, Varna, Bulgaria; 2 Department of General and Clinical Pathology, Forensic Medicine and Deontology, Medical University – Varna "Prof. Dr. Paraskev Stoyanov", Varna, BGR

**Keywords:** psoas muscle, psoas minor, morphometric study, psoas minor morphology, bulgaria

## Abstract

Introduction

The psoas muscle group is part of the posterior abdominal wall and is comprised of long muscles – major, minor, and tertius. Out of those, only the psoas major muscle is an obligatory muscle present in all individuals. The psoas minor muscle (PMM) originates as vertical fascicles inserted in the bodies of the last thoracic and first lumbar vertebrae and inserting into the iliopectineal eminence. The muscle provide flexion of the lumbar spine in a limited fashion. The aim of the study was to establish the frequency of the muscle in the Bulgarian population.

Materials and methods

This study was carried out in the Department of Anatomy and Cell Biology, Medical University, Varna, Bulgaria, by Dr. Paraskev Stoyanov in November 2017, on a total of 10 cadavers. The length, width, and circumference of the muscles were measured. The collected data were interpreted in a descriptive manner.

Results

The PMM was present in six out of ten cadavers (60%). Out of those six cadavers, the muscle was bilateral in three, unilateral on the left side in one, and unilateral on the right side in two cadavers. The average length of the muscle was 19.66 cm (range:14.4 cm - 21.7 cm), average width was 1.73 cm (range: 1.0 cm - 3.2 cm ) and average circumference was 3.48 cm (range: 1.7 cm - maximum 5.6 cm). The male to female ratio of cadavers with a PMM was 1:1.

Conclusion

The frequency of the muscle's variations considering its presence in the Bulgarian population (60%) is higher when compared to its presence in the Indian population (36.67%), virtually identical to the Brazilian population (59%), and lower than that reported in the US (65.6%). The morphometric analyses of the different populations showed a shorter psoas minor in the Bulgarian population.

## Introduction

The psoas muscle group is part of the posterior abdominal wall and is comprised of long muscles – major, minor, and tertius [1]. Out of those, only the psoas major muscle is an obligatory muscle present in all individuals [2-6]. The psoas minor muscle (PMM) originates as vertical fascicles inserted in the bodies of the last thoracic and first lumbar vertebrae and are inserted into the iliopectineal eminence [6]. The muscle’s actions provide for flexion of the lumbar spine in a limited fashion. The aim of the study was to establish the frequency of the muscle in the Bulgarian population.

## Materials and methods

The study was carried out in the Department of Anatomy and Cell Biology, Medical University, Varna, Bulgaria, by Dr. Paraskev Stoyanov, in November 2017, on cadavers used for teaching purposes. The research was based on the standard dissection of the posterior abdominal wall on a total of 10 cadavers, selected on a random basis. In the first step, a statistical approach was undertaken to establish the presence of the muscle, together with morphological variations. In the second step, the length of the muscle and its tendon and the width and circumference of the belly at the widest point, were measured. The collected data was interpreted in a descriptive statistical manner.

## Results

The muscle was present in six of the ten cadavers. However, out of those six cadavers, a bilateral PMM was found in three. A unilateral muscle on the left side was found in one cadaver and a unilateral PMM was found on the right side of two cadavers (Table 1).

**Table 1:** Morphometric data

№	Gender	Psoas minor present	Length		Width			Circumference	
			Left	Right	Left	Right		Left	Right
1	Female	-	-		-			-	
2	Male	Bilateral	19.2 cm	20.1 cm	1.4 cm	3.2 cm		3.5 cm	5.2 cm
3	Male	Left	19.5 cm	-	3.2 cm	-		5.6 cm	-
4	Female	Right	-	21.7 cm	-	1.5 cm		-	2.4 cm
5	Male	-	-		-			-	
6	Male	Bilateral	16.4 cm	14.4 cm	1 cm		1.8 cm	2.2 cm	4.6 cm
7	Female	Right	-	21.2 cm	-		2.1 cm	-	3.4 cm
8	Male	-	-		-			-	
9	Female	-	-		-			-	
10	Female	Bilateral	20.6 cm	17.8 cm	1 cm	1.4 cm		2.7 cm	1.7 cm

All specimens presented with the standard muscle morphology - the belly of the muscle originated from the last thoracic and first lumbar vertebrae and its tendon inserted into the illiopectineal eminence. No morphological variants of the muscle such as a split muscle belly or tendon were observed.

The mean length of the muscle was 19.66 cm (range 14.4 cm - 21.7 cm), mean width was 1.73 cm (range 1 cm - 3.2 cm), and mean circumference was 3.48 cm (range 1.7 cm - 5.6 cm). The male to female ratio of cadavers with a PMM, regardless of whether bilateral or unilateral, was 1:1.

## Discussion

The PMM shows varying lengths, widths, and circumferences in different populations. As already mentioned, the mean length in our cohort was 19.66 cm and mean width was 1.73 cm. For comparison, Joshi et al. found that in one population in India, the PMM was present in 30% with a mean length of 23.75 cm and a mean width of 1.32 cm. A second study from India, by Ohja et al., found a PMM in 26.66% of the cases with a mean length of 22.12 cm [4,7]. In Brazil, Farias et al. found the muscle to be present in 59% of the population with a mean length of 23.93 cm and a mean width of 1.71 cm [8]. In the US, Neumann et al. found the PMM to be present in 65.6% of the population with a mean length of 23.85 cm [5].

When compared together, the individual results show that despite variances in the reported figures, the Indian population has the longest PMM and the Bulgarian one has the shortest. The mean width of the muscle, measured at the widest point of the belly, on the other hand, showed no significant difference in the Bulgarian, Brazilian, and Indian populations.

The major clinical implications of the PMM are those of the so-called psoas minor syndrome, a subtype of the more common psoas syndrome [6,9]. The psoas minor syndrome is a multifactual condition, often caused by a tense muscle and tendon, resulting in the compression of the retroperitoneal neurovascular structures, often presenting as chronic lower abdominal or lumbar pain. Other reasons for the condition include primary and metastatic neoplasms, as well as idiopathic factors. The changes in the muscle tension result in chronic pain, often localized in the area of compression or tenderness. Patients with this syndrome complain of pain predominantly localized in the corresponding iliac fossa, which is aggravated by palpation. In cases of chronic lumbar pain, palpation of the corresponding lower abdomen results in increased pain. Due to the presentation, the syndrome may be misinterpreted as appendicitis if present on the right side, or as extrauterine pregnancy [9]. Even if correctly diagnosed, the psoas syndrome is often difficult to treat, as the pain requires both physical rehabilitation and opioid analgesics in some of the cases [10]. 

## Conclusions

The PMM is a greatly variable muscle, as seen in the individual metrics in the Bulgarian population and the reports from other populations. In Brazil, the PMM has an incidence of 59%, and the USA, 65%. However, there is great variance in the reported figures from the two Indian populations where the PMM was present in only 30% and 26.66% respectively. The morphometric analyses of the different aforementioned populations showed a shorter PMM in the Bulgarian population, with nearly identical figures for muscle width across all populations, although individual muscle belly size varied greatly in all populations.
